# *Ciboria carunculoides* Suppresses Mulberry Immune Responses Through Regulation of Salicylic Acid Signaling

**DOI:** 10.3389/fpls.2021.658590

**Published:** 2021-04-06

**Authors:** Zhiyuan Lv, Lijuan Hao, Bi Ma, Ziwen He, Yiwei Luo, Youchao Xin, Ningjia He

**Affiliations:** State Key Laboratory of Silkworm Genome Biology, Southwest University, Chongqing, China

**Keywords:** mulberry sclerotial disease, necrotrophic pathogen, proanthocyanidins, salicylic acid, effector, PR1

## Abstract

*Ciboria carunculoides* is the dominant causal agent of mulberry sclerotial disease, and it is a necrotrophic fungal pathogen with a narrow host range that causes devastating diseases in mulberry fruit. However, little is known about the interaction between *C. carunculoides* and mulberry. Here, our transcriptome sequencing results showed that the transcription of genes in the secondary metabolism and defense-related hormone pathways were significantly altered in infected mulberry fruit. Due to the antimicrobial properties of proanthocyanidins (PAs), the activation of PA biosynthetic pathways contributes to defense against pathogens. Salicylic acid (SA) and jasmonic acid (JA) are major plant defense hormones. However, SA signaling and JA signaling are antagonistic to each other. Our results showed that SA signaling was activated, while JA signaling was inhibited, in mulberry fruit infected with *C. carunculoides*. Yet SA mediated responses are double-edged sword against necrotrophic pathogens, as SA not only activates systemic acquired resistance (SAR) but also suppresses JA signaling. We also show here that the small secreted protein CcSSP1 of *C. carunculoides* activates SA signaling by targeting pathogenesis-related protein 1 (PR1). These findings reveal that the infection strategy of *C. carunculoides* functions by regulating SA signaling to inhibit host defense responses.

## Introduction

Mulberries are the nutritious, delicious, and wholesome fruits from mulberry trees, which grow in a variety of areas around the world (Chan et al., 2016). Many nutrients levels in mulberry are superior to other fruits, such as the levels of iron, potassium, calcium, riboflavin, vitamin C, vitamin K, dietary fiber, and a wide range of organic compounds, including resveratrol, anthocyanins, and various polyphenolic compounds (Liang et al., 2012). Nowadays, the mulberry industry is booming in China due to its excellent dual-use value for medicine and food. However, the yield of mulberry is severely affected by mulberry sclerotial disease, a devastating fungal disease. Almost all *Morus alba* L. varieties are susceptible to mulberry sclerotial disease, and *M. alba* is native and widely cultivated in China. The current prevention and control of mulberry sclerotial disease is costly and laborious, and the results are often unsatisfactory.

There are three kinds of pathogens that can cause mulberry sclerotial disease, *Ciboria carunculoides*, *Scleromitrula shiraiana*, and *Ciboria shiraiana* (Siegler and Jenkins, 1923; Whetzel and Wolf, 1945; Hong et al., 2007). *C. carunculoides* is the dominant pathogen of mulberry sclerotial disease in China and many other countries (Whetzel and Wolf, 1945; Sultana et al., 2013; Dai et al., 2019). *S. shiraiana*, however, is found only in a relatively small region of Southwest China (Lü et al., 2017). In contrast, *C. shiraiana* is often found in a subset of mulberry growing areas (Sultana and Kim, 2016; Bao et al., 2020). These three fungi are pathogens with a narrow host range, because to date there have been no reports about these pathogens infecting plants other than mulberry under natural conditions. However, *Ciboria* genus pathogens are difficult to propagate on artificial media (Hong et al., 2007; Sultana et al., 2013). This has been one of the major obstacles to detailed a characterization of *Ciboria* and an analysis of its pathogenic mechanism.

Plant innate immune response includes pattern-triggered immunity (PTI) and effector-triggered immunity (ETI) (Jones and Dangl, 2006). PTI is an ancient and basic form of plant immunity that provides limited immunity to host-adapted pathogens (Böhm et al., 2014). However, the robustness of the plant immune signaling network is higher during ETI than PTI (Tsuda et al., 2009). The induced defense response in plants depends upon the lifestyle and offensive strategy of the plant pathogen (Mengiste, 2012; Pandey et al., 2016). The activation of the plant defense response leads to the accumulation of phytohormones such as salicylic acid (SA), and jasmonic acid (JA) and ethylene (Glazebrook, 2005; Yu et al., 2017). SA and JA and their derivatives are recognized as major defense hormones (Pieterse et al., 2012). SA is involved in the defense response to biotrophic and hemibiotrophic pathogens, while JA is the primary hormone involved in the response to necrotrophic pathogens. These two pathways are mutually antagonistic (Robert-Seilaniantz et al., 2011). The mutual antagonism of SA and JA is exploited by some pathogens, which produce phytohormones or phytohormone mimics. Many strains of *Pseudomonas syringae* produce the phytotoxin coronatine, which is a mimic of bioactive JA-isoleucine and suppresses SA-dependent defenses, thereby promoting susceptibility of the plant to the pathogens (Brooks et al., 2005; Fonseca et al., 2009). The necrotrophic pathogen *Botrytis cinerea* produces an exopolysaccharide, which activates the SA signaling pathway and suppresses the JA signaling pathway, thereby overcoming host immunity and promoting disease (El Oirdi et al., 2011). The biotrophic oomycete *Hyaloperonospora arabidopsidis* secretes nuclear-localized effector HaRxL44, which interacts with Mediator subunit 19a (MED19a) to attenuate the expression of SA-dependent genes and enhance susceptibility to this pathogen (Caillaud et al., 2013). Moreover, cerato-platanin protein SsCP1 from *Sclerotinia sclerotiorum* targets plant pathogenesis-related protein 1 (PR1) and triggers plant SA-related defense responses (Yang et al., 2018).

Proanthocyanidins (PAs) are secondary metabolites that are abundant in plants and have antimicrobial activity (Yuan et al., 2012). PA biosynthesis is often up-regulated after pathogen infection (Miranda et al., 2007; Guidarelli et al., 2011). For example, transgenic plants with higher PAs content showed enhanced resistance to fungal pathogens than control lines (Wang et al., 2017; Xin et al., 2020). PAs and other secondary metabolites derived from flavonoids are likely to be active substances that induce broad-spectrum resistance, yet their anti-pathogenic effects are very limited in most plants. This is often because many pathogens can easily overcome the obstruction of these secondary metabolites (Chen et al., 2019).

Lately, increasing attention has been paid to mulberry sclerotial disease. Unfortunately, studies on the molecular mechanisms of mulberry resistance to mulberry sclerotial disease and the pathogenesis of pathogens are scarce. In this study, we conducted a comparative transcriptomic analysis of mulberry fruit infected or mock-infected with *C. carunculoides* using RNA-seq. In several defense-associated pathways and gene families, such as flavonoid biosynthesis, phenylpropanoid biosynthesis, plant hormone signal transduction, and chitin recognition genes, the number of differentially up-regulated genes was significantly enriched. PAs biosynthesis genes were activated in infected mulberries. Tobacco plants overexpressing mulberry anthocyanidin reductase (ANR) and leucoanthocyanidin reductase (LAR) showed not only increased content of PAs but also increased accumulation of catechin and epicatechin, respectively. We further demonstrated that PAs, not catechin or epicatechin, were effective antifungal ingredients. We also found that pathogen activates mulberry SA signaling and antagonizes JA signaling. The role of SA in plant defense against necrotrophic pathogens is complex. Not only did SA antagonize JA signaling and induce cell death, but the systemic acquired resistance (SAR) induced by SA also had a certain inhibitory effect on the colonization and spread of necrotrophic pathogens. *C. carunculoides* is a necrotrophic pathogenic fungus. JA is mainly involved in resistance to necrotrophic pathogens. The activation of SA signaling in infected mulberry is more conducive to the spread of pathogen. We also showed that a small secreted protein, CcSSP1, from *C. carunculoides* induced the activation of SA signaling by targeting PR1. This study not only improved our collective understanding of the mechanism of effectors in regulating pathogenicity but also showed the complex role of SA in plant defense against necrotrophic pathogens.

## Materials and Methods

### Plant Material and *C. carunculoides* Inoculation

The *M. alba* L. cultivar Hongguo II was grown in the field of the Mulberry Breeding Center of Southwest University in Chongqing, China. Hongguo II is susceptible to *C. carunculoides*, as are other white mulberry cultivars. *C. carunculoides* is an ascomycete fungus, and its ascospores are the only source of primary infection. The ascospores of *C. carunculoides* were collected from mature fruit bodies in a mulberry orchard. Ascospores were then diluted in sterile water to 5 × 10^6^/ml and brushed on the decaying female flowers with a small brush. Non-inoculated (brushed with sterile water) replicated plants were included in assays as controls.

To confirm that inoculated samples were effectively infected, all infected samples were collected when disease symptoms appeared after inoculation. All samples (inoculated and non-inoculated) were covered with bagging paper. Three biological replicates of each mulberry fruit sample were collected at three different stages of the disease. Stage 1, stage 2, and stage 3 represent the initial, middle, and middle-late stages of mulberry infection, respectively. Only a few drupelets of mulberry were infected at the stage 1. At the initial stage, the infected drupelets appeared chlorotic yellowish, and the tops of the perianth and ovary appeared brownish red (Supplementary Figure 1). The pathogen proliferated and infected more drupelets at stage 2, but did not appear as white and mummified drupelets. At stage 3, some early infected drupelets became mummified and enlarged. With the development of disease, the whole fruit became white and swollen.

### RNA Extraction, Library Preparation, and Sequencing

Total RNA was isolated using the RNAprep pure Plant Kit (Tiangen Biotech, Beijing, China) from *C. carunculoides* infected and healthy mulberry fruits according to the manufacturer’s instructions. Then the total RNA was treated with RNase-free DNase I (Promega, WI, United States) for 30 min at 37°C to remove residual genomic DNA. RNA quality was assessed on an Agilent 2100 Bioanalyzer (Agilent Technologies, CA, United States) and checked using RNase free agarose gel electrophoresis. RNA samples were subjected to library construction using a TruSeq^TM^ RNA Sample Preparation Kit from Illumina (San Diego, CA, United States) and 5 μg of total RNA for each library. The library preparations were sequenced using the HiSeq 2,000 sequencing system (Illumina) in the paired-end mode by Gene *Denovo* Biotechnology Co., (Guangzhou, China). The raw reads were cleaned by removing adaptor sequences, empty reads, and low-quality sequences (reads with unknown sequences “N” or less than 25 bp). The clean reads were then assembled into non-redundant transcripts using Trinity (Grabherr et al., 2011).

### Transcriptomic Data Analysis

To annotate unigenes, we used the BLASTx program^1^ with an *E*-value threshold of 1e-5 and the NCBI non-redundant protein (Nr) database^2^, the Swiss-Prot protein database^3^, the Kyoto Encyclopedia of Genes and Genomes (KEGG) database^4^, and the COG/KOG database^5^. Gene differential expression analysis was performed using DESeq2 software (Love et al., 2014). The genes with a false discovery rate (FDR) below 0.05 and an absolute fold change ≥2 were considered differentially expressed genes. DEGs were then subjected to enrichment analysis for GO functions and KEGG pathways.

### Antifungal Tests With Catechin, Epicatechin, and PAs

As the *C. carunculoides* cannot be cultivated on artificial medium yet, another pathogen of mulberry sclerotial disease, *S. shiraiana* (strain SX-001), was used in antifungal tests. *S. shiraiana* was cultivated on PDA medium containing catechin, epicatechin, catechin and epicatechin, PAs (C_30_H_26_O_13_), and all three, respectively. The working concentrations of catechin, epicatechin and PAs were 600, 100 μg/ml, and 10 mg/ml, respectively. The colonies were measured and photographed after 13 days of incubation at 24°C.

### Quantification of SA

Plant samples were ground into powder with liquid nitrogen, weighed (*c*. 100 mg), and resuspended in 80% (v/v) methanol at 4°C for 12 h. The samples were next sonicated in an ice-water bath for 30 min, and then centrifuged at 13,000 × *g* for 5 min at 4°C. The SA in the supernatant was enriched with an SPE C18 column (Welch Materials, Inc., Shanghai, China). A SA standard was diluted using different concentration gradients with methanol. Samples were analyzed with a Hybrid Quadrupole-TOF LC/MS/MS Mass Spectrometer (Shimadzu Corporation, Kyoto, Japan). The separation was done with a Shimadzu InerSustain C18 column (100 mm × 2.1 mm, 2 μm). The injection volume was 10 μL.

### Botrytis Cinerea Inoculation and SA Treatment

The *B. cinerea* isolate MM1 was isolated from mature mulberry fruit. The isolate was routinely sub-cultured on PDA at 24°C to maintain vigor and purity and was preserved for a long time in glass tubes containing PDA at 4°C. *Nicotiana benthamiana* plants (4 to 6 weeks old) were injected with different concentrations of SA (10, 100 μM, or 2 mM) using needleless syringes. Then *B. cinerea* isolates MM1 cultured on PDA at 24°C for 4 days were inoculated into the plants. *Nicotiana* leaf lesions were measured 4 days after inoculation. Compared with other mulberry cultivars, long-fruit mulberry was not susceptible to mulberry sclerotial disease. SA (200 μM) and 3HBA (200 μM) were sprayed on the long-fruit mulberries with a low disease index (each fruit had no more than three diseased drupelets). All treated long-fruit mulberries were wrapped in paper bags. After 1 week, the disease index of the diseased fruit in each treatment group was determined.

### Disease Index of Diseased Long-Fruit Mulberry

The disease index of diseased long-fruit mulberry was rated on a scale from 0–9. 0, healthy fruit; 1, <5% of fruit area infected (less than three small drupelets were infected without spreading); 3, 5–15% of fruit area infected (*c.* 10 small drupelets were infected, and only one place where the infected fruits spread); 5, 15–30% of fruit area infected (infected fruits spread in more than two places); 7, 30–50% of fruit area infected (infected fruits spread significantly in many places); 9, >50% of fruit area infected (Supplementary Figure 5).

### Prediction of Fungal Effector Proteins

First, fungal secretory proteins were predicted using SignalP 5.0 (Almagro Armenteros et al., 2019). Then, secreted proteins containing a transmembrane domain were removed using TMHMM 2.0 (Krogh et al., 2001). To predict effectors, Big-PI Fungal Predictor was used to filter out secretory proteins that contained putative glycophosphatidylinositol membrane-anchoring domain (Eisenhaber et al., 2004). Finally, EffectorP 2.0 was used to predict potential effectors in the remaining secretory proteins (Sperschneider et al., 2018).

### Transient Expression Analysis of Putative Effectors in *N. benthamiana*

Putative effector genes were cloned into pGR107. The GR107 vector was linearized by digestion with *Cla*I and *Sal*I. Then, each construct was transformed into the *Agrobacterium tumefaciens* strain GV3101 containing the helper plasmid pJIC SA_Rep. Infiltration experiments were performed on 4- to 6-week-old *N. benthamiana* plants using needleless syringes as described previously (Lv et al., 2018). GFP and the pro-apoptotic mouse protein BAX served as a negative control and positive control, respectively. Cell death symptoms were photographed at 6 d after infiltration. The results are representative of three biological replicates.

### Y2H and GST Pull-Down Assays

A Y2H assay was performed using the Matchmaker Gold yeast two-hybrid system as described previously (Lü et al., 2017). *CcSSP1* was introduced into pGBKT7 as bait, and other candidate genes were introduced into pGADT7 as prey. For glutathione S-transferase (GST) pull-down assays, pGEX-4T-1 and pET32a vectors were used for the preparation of CcSSP1 and MaPR1, respectively. GST-CcSSP1 and His-MaPR1 were expressed in the *Escherichia coli* strain BL21 (DE3). Protein induction was performed by addition of 0.5 mM isopropyl β-D-1-thiogalactopyranoside (IPTG) at 37°C for 3 h. Then, 1 ml of GST and GST-CcSSP1 sonicated soluble supernatant was incubated with 30 μl PureCube Glutathione MagBeads (Cube Biotech) at 4°C for 1 h. The supernatant was then removed, and MagBeads were added to the supernatant expressing His-MaPR1 and incubated at 4°C for 1 h. GST fusion proteins were eluted with reduced glutathione. Immunoblot was used for detection of His-MaPR1 and GST fusion proteins with a-His and a-GST antibodies, respectively. The primer sequences are listed in Supplementary Table 1.

### Statistical Analysis

All data were analyzed using Student’s *t*-test or one-way ANOVA with SPSS 18.0. The values represented as means ± standard deviation (SD).

### Accession Code

The raw data in this paper have been deposited in the Genome Sequence Archive in National Genomics Data Center, Beijing Institute of Genomics (China National Center for Bioinformation), Chinese Academy of Sciences, under accession number CRA003673 that are publicly accessible at https://bigd.big.ac.cn/gsa.

## Results

### Transcriptome Sequencing of Diseased Mulberry Fruits

To identify candidate genes associated with innate immunity in mulberry infected with *C. carunculoides*, the transcriptome of diseased mulberry fruit from early (Stage 1), interim (Stage 2), and middle-late (Stage 3) infection were sequenced on an Illimina Hiseq 2000 (Supplementary Figure 1). A total of 65,653 transcripts were expressed in at least one treatment group. There were only 35 differentially expressed genes (DEGs) in stage 1 infected fruit. Due to the limited scale of infection at this stage, the DEGs in stage 1 may have been diluted by the higher proportion of uninfected tissues in these samples. The number of DEGs from mulberry increased significantly in stage 2 and stage 3 to 3628 and 4669, respectively (Supplementary Figure 2).

### DEGs Related to Plant Immunity

**FIGURE 1:**
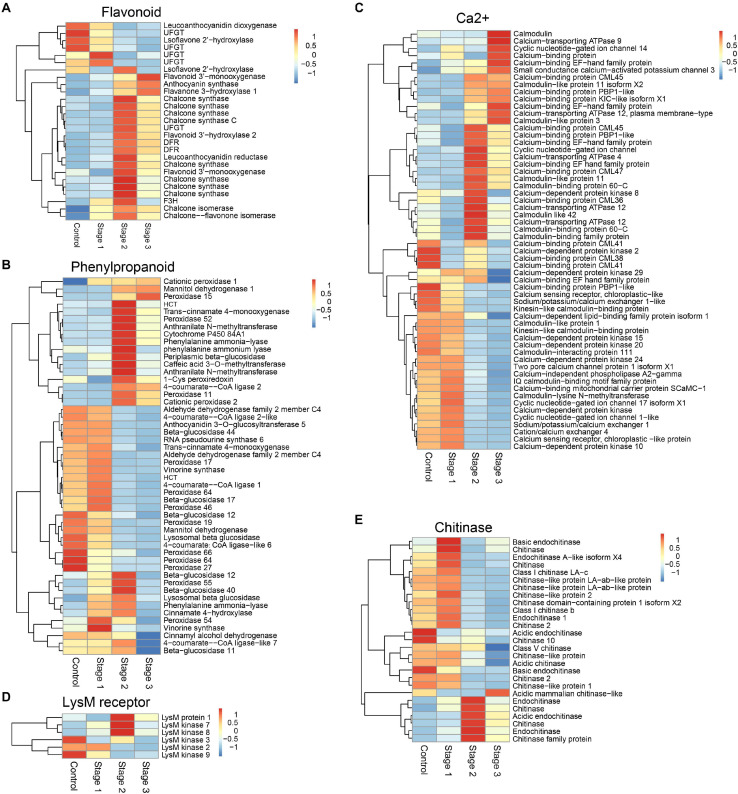
DEGs associated with different defense-associated biosynthetic pathways or gene families. **(A–B)** DEGs in flavonoid biosynthesis and phenylpropanoid biosynthesis pathways. **(C)** DEGs related to calcium signal transduction. **(D)** DEGs encoding lysM motif receptor. **(E)** DEGs encoding chitinase. DFR, bifunctional dihydroflavonol 4-reductase/flavanone 4-reductase; UFGT, UDP-glucose flavonoid 3-O-glucosyltransferase; F3H, flavanone 3-hydroxylase; HCT, hydroxycinnamoyl-Coenzyme A shikimate/quinate hydroxycinnamoyltransferase.

KEGG pathway analysis showed that the DEGs in the flavonoid biosynthesis, phenylpropanoid biosynthesis, photosynthesis, plant-pathogen interaction, biosynthesis of secondary metabolites, photosynthesis-antenna proteins, plant hormone signal transduction, and metabolic pathways were enriched in stage 2 infected fruits. The DEGs in metabolic pathways, DNA replication, flavonoid biosynthesis, and biosynthesis of amino acids were enriched in stage 3 (Supplementary Figure 3). Among these pathways, flavonoid biosynthesis and phenylpropanoid biosynthesis were two important pathways that have been shown to be related to plant immunity (Figure 1). Calcium, as an essential second messenger, plays an important role in regulating plant immunity-related pathways. A large number of genes related to calcium signal transduction were differentially expressed in diseased mulberries (Figure 1C). Mulberry sclerotial disease is a fungal disease, so the genes encoding the receptor protein and chitinase that recognize fungal chitin were analyzed further. There were six genes encoding chitinases and three genes encoding chitin receptors that were significantly up-regulated in stage 2 infections (Figures 1D–E). One of these, LysM protein 1, is a homolog of the chitin recognition receptor CEBiP (chitin elicitor-binding protein).

### PAs Inhibit the Vegetative Growth of *Scleromitrula shiraiana*

**FIGURE 2:**
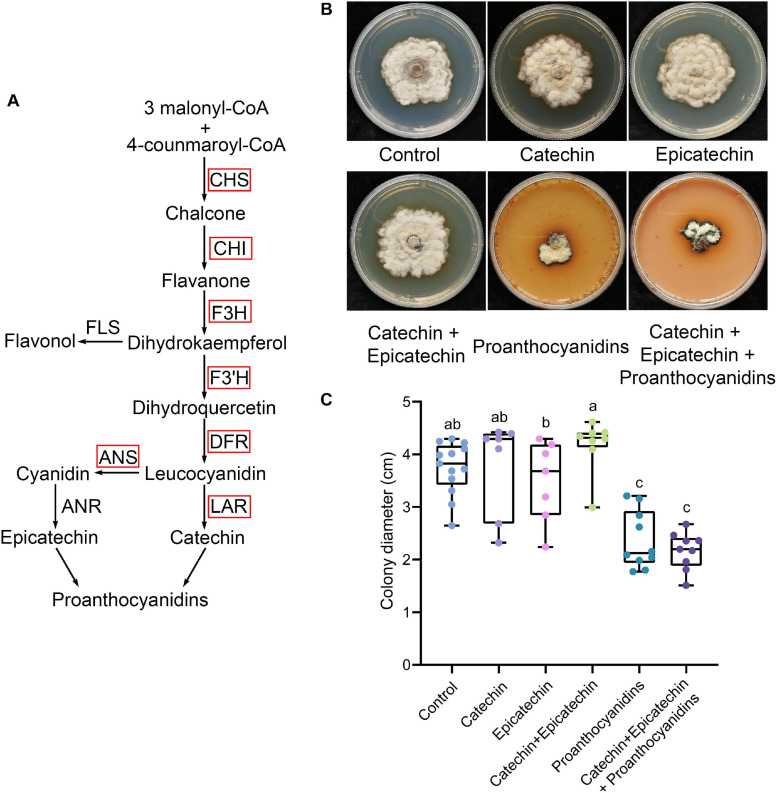
Proanthocyanidins inhibit the vegetative growth of *Scleromitrula shiraiana*. **(A)** Most of the key genes in the biosynthetic pathway of proanthocyanidins in infected mulberries were up-regulated. CHS, chalcone synthase; CHI, chalcone isomerase; F3H, flavanone 3–hydroxylase; F3′H, flavonoid 3′–hydroxylase; DFR, dihydroflavonol 4-reductase; LAR, leucoanthocyanidin reductase; ANS, anthocyanin synthase. **(B–C)** The effects of proanthocyanidins, catechin and epicatechin on the vegetative growth of *S. shiraiana*. *S. shiraiana* is another pathogen of mulberry sclerotial disease. Colony photographs and colony diameter measurements were taken on PDA medium at 25°C after 13 days. Control indicates growth without any treatment. Different letters (a, b) indicate statistical differences (*P* < 0.05) (*n* > 7, one-way ANOVA with *post hoc* Duncan’s test). The experiments were repeated at least three times.

PAs are the main phenolic compounds related to plant defense. Most of the PA biosynthesis-related genes in diseased mulberries were up-regulated (Figures 1A, 2A). Our previous studies showed that *N. tabacum* overexpressing ANR and LAR of *Morus notabilis* had enhanced resistance to *B. cinerea* (Xin et al., 2020). The content of catechin, epicatechin, and PAs in tobacco overexpressing *MnANR* and *MnLAR* were also shown to be significantly increased (Xin et al., 2020). Thus, we wondered if catechin, epicatechin, and PAs are resistant to necrotrophic pathogens. To test this, *S. shiraiana*, another pathogen of mulberry sclerotial disease, was cultured on PDA medium supplemented with catechin, epicatechin, or PAs. The results showed that only the addition of PAs significantly inhibited the vegetative growth of *S. shiraiana* (Figures 2B,C). The mixed addition of catechin and epicatechin could not inhibit the growth of *S. shiraiana* (Figures 2B,C). A mixture of catechin, epicatechin, and PAs had the same inhibitory effect on *S. shiraiana* as the addition of PAs alone (Figures 2B,C).

### The Biosynthesis and Signal Transduction Genes Related to Salicylic Acid and Jasmonic Acid in Diseased Fruits Had Opposite Expression Trends

**FIGURE 3:**
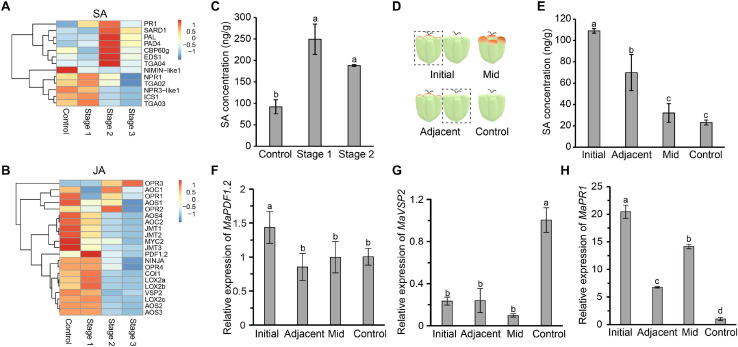
The salicylic acid signaling pathway is induced in infected mulberry fruits. **(A)** The SA immune signaling pathway was activated in diseased mulberries. **(B)** The JA immune signaling pathway was inhibited in diseased mulberries. **(C)** The content of SA in diseased mulberry fruits in stage 1 and stage 2. **(D)** Schematic diagram of mulberry drupelet. Initial, initial infected drupelet with slight symptoms; Mid, diseased drupelet in the middle stage; Adjacent, uninfected drupelet adjacent to the initial infected drupelet; Control, uninfected drupelet from healthy mulberry. **(E)** The content of SA in different drupelets. **(F–G)** The expression of the marker genes *MaPDF1.2* and *MaVSP2* in the JA immune signaling pathway in different drupelets. **(H)** The expression of the marker gene *MaPR1* in the SA immune signaling pathway in different drupelets. *MaActin* was used as an internal reference gene. Different letters (a, b) indicate statistical differences (*P* < 0.05) (*n* = 3, one-way ANOVA with *post hoc* Duncan’s test). The q-PCR analyses were repeated twice, and two mulberry fruits were used for each sample.

Salicylic acid and JA are two important plant defense-related hormones that instill resistance to biotrophic pathogens and necrotrophic pathogens, respectively. SA- and JA-mediated defense pathways are antagonistic to each other. SA biosynthesis and signal transduction pathways are activated in diseased mulberries, while the opposite is true for JA (Pieterse et al., 2012). *PR1*, a marker gene of the SA signaling transduction pathway, was significantly up-regulated in the three stages of disease (Figure 3A). Negative regulatory genes, such as *NIMIN-like1* and *NPR3-like1*, were down-regulated (Figure 3A). Most of the key genes in the JA biosynthesis and signal transduction pathways were expressed at higher levels in stage 1 diseased fruit. The expressions of the marker genes *PDF1.2* and *VSP2* in the JA signaling pathway were also significantly increased (Figure 3B). However, the JA signaling pathway was inhibited in stage 2 and stage 3. Correspondingly, SA signaling was activated at these times.

Since the SA biosynthesis and signaling pathways in diseased fruit were activated, we next asked if the content of SA in diseased fruit increased. As diseased fruit tissue already appeared necrotic in stage 3, we only determined the SA content in diseased fruit from the stage 1 and stage 2. The results showed that the level of SA in diseased fruit was significantly higher than that of uninfected mulberries, and the SA content in diseased fruit in stage 1 was the highest (Figure 3C). Stage 1 is the initial stage of the disease. In this initial stage, the pathogen may antagonize the JA signaling pathway by activating host SA biosynthesis. In order to test this hypothesis, we further tested the SA content of drupelets. The SA content of drupelets in the initial stage of infection was significantly higher than that of healthy drupelets (Figures 3D,E). Interestingly, the SA level of the uninfected drupelets adjacent to the initial diseased drupelets was also significantly higher than that of healthy drupelets (Figure 3E). However, the SA content of drupelets in the middle stage of the disease (when tissue necrosis had not yet occurred) was equivalent to that of healthy drupelets (Figure 3E). Furthermore, the transcription levels of marker genes of the SA and JA signaling pathways in these drupelets were detected. The expression level of *MaPDF1.2* was similar in adjacent, mid-stage and control drupelets, but it was significantly up-regulated only in the initial stage of infection (Figure 3F). The expression level of *MaVSP2* was significantly lower in the initial, mid-stage and adjacent drupelets than in controls (Figure 3G). However, correspondingly, the expression of *MaPR1* was significantly higher in initial, mid-stage, and adjacent drupelets than in controls (Figure 3H). These findings were consistent with our transcriptome data (Figures 3A,B). This indicated that the SA signaling of infected mulberry was activated, while JA signaling may be inhibited, upon infection.

### SA Plays a Complex Role in Defending Against Necrotrophic Pathogens

**FIGURE 4:**
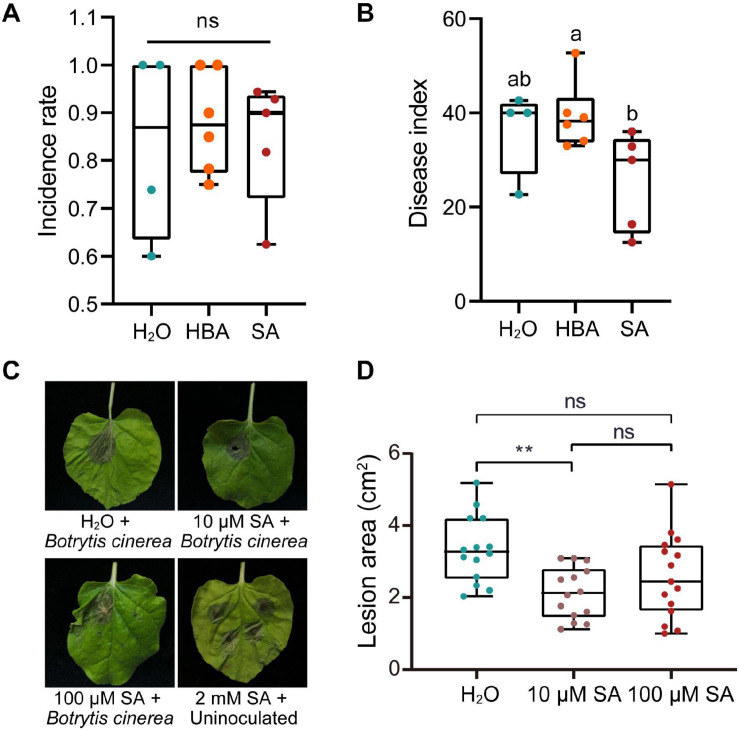
The effect of salicylic acid on the infection of plants by pathogens. **(A)** The incidence rate of mulberry fruit used for spraying with SA or HBA. The incidence rate and disease index of diseased mulberry in all groups were consistent (*n* ≥ 4). HBA, 3-hydroxy benzoic acid. H_2_O, Control group, sprayed with double distilled water. **(B)** The effect of spraying with SA or HBA on the disease index of infected mulberry fruits. The disease index of the diseased fruit was analyzed 7 days after spraying. Different letters (a, b) indicate statistical differences (*P* < 0.05) (*n* ≥ 4, one-way ANOVA with *post hoc* Duncan’s test). **(C)** The effect of injection with different of concentrations of SA on *Nicotiana benthamiana* with *Botrytis cinerea* infection. Tobacco leaves infected with *B. cinerea* were photographed at 4 days after inoculation. **(D)** Quantitative measurement of lesion size on tobacco leaves inoculated with *B. cinerea*. Asterisks indicate a statistically significant difference (**P < 0.01) according to Student’s *t*-test. Experiments were repeated at least three times.

SA not only activates local resistance, but also induces SAR in plants. The application of exogenous SA can induce a host’s resistance to various pathogens, which is likely to activate the host’s SAR. We then sought to verify whether exogenous SA could inhibit the proliferation of pathogen in mulberries. Long-fruit mulberries with fewer than three diseased drupelets were sprayed with SA (200 μM) or its inactivating analog, 3-hydroxy benzoic acid (3HBA) (200 μM). After 1 week, the disease index of long-fruit mulberries sprayed with SA was lower than that of those sprayed with 3HBA and double distilled water (Figures 4A,B). This indicated that the pre-established defense response induced by SA prevented the proliferation of pathogens in diseased fruit.

However, SA induces programmed cell death in plants and plays a role in plant defenses against biotrophic pathogens, and the programed cell death of plants is beneficial to the colonization and infection of necrotrophic fungi. Thus, we wondered what the response of plants pretreated with different concentrations of SA to necrotrophic fungi would be. Injection of 2 mM SA into the leaves of *N. benthamiana* caused obvious cell death (Figure 4C). However, injection of 10 or 100 μM SA did not induce tobacco cell death, and 10 μM SA significantly inhibited the expansion of necrotrophic *B. cinerea* (Figures 4C,D).

### An Effector Protein of *C. carunculoides* Contributes to the Activation of SA Signaling

**FIGURE 5:**
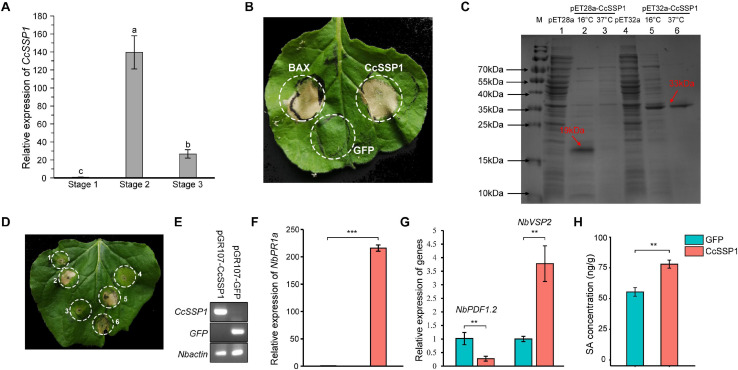
CcSSP1 induces tobacco cell death and activates the salicylic acid signaling. **(A)** *CcSSP1* was up-regulated during the process with *Ciboria carunculoides* infecting mulberries. *CcActin* was used as an internal reference gene. Letters a, b indicate statistical differences, *n* = 3, *P* < 0.05. **(B)** CcSSP1 induced cell death in *Nicotiana benthamiana*. Leaves of *N. benthamiana* were transformed with indicated constructs by agro-infiltration. Photographs were taken 6 d post agroinfiltration. **(C)** Prokaryotic expression of CcSSP1. The recombinant CcSSP1 is marked with arrows. **(D)** The supernatant of prokaryotically expressed CcSSP1 induced cell death in *N. benthamiana*. 1, supernatant from empty pET28a vector; 2–3, supernatant from pET28a-CcSSP1 induced at 16 and 37°C, respectively; 4, supernatant from empty pET32a vector; 5–6, supernatant from pET32a-CcSSP1 induced at 16 and 37°C, respectively. Photographs were taken 5 days after infiltration with recombinant proteins. Results are representative of three biological replicates. **(E)** Semi-quantitative measurement of the transcription of *CcSSP1* and *GFP* transiently expressed in *N. benthamiana*. **(F)** After transient expression of *CcSSP1* and *GFP*, the relative expression of the SA defense signal marker *NbPR1a* in *N. benthamiana*. **(G)** After transient expression of *CcSSP1* and *GFP*, the relative expression of the JA signaling pathway markers *NbPDF1.2* and *NbVSP2* in *N. benthamiana*. The transcript levels were monitored by quantitative reverse transcription PCR. *NbActin* was used as an internal reference gene. The q-PCR analyses were repeated at least three times. **(H)** Measurement of the SA content in *N. benthamiana a*fter transient expression of *CcSSP1* or *GFP.* The leaf samples in (E–H) were collected 2 days after Agrobacterium infiltration. Each treatment contains three technical replicates, and each technical replicate sample uses two leaves. Asterisks indicate a statistically significant difference (**P < 0.01, ***P < 0.001) based on Student’s *t*-test.

Plant SA signaling is probably induced by necrotrophic pathogens and is used to antagonize JA signaling. A specific effector protein is thus a crucial weapon in the early stage of pathogen infection. The SA signaling of mulberry fruit may be induced by such an effector protein. Based on our transcriptome data from diseased fruit, we identified an effector whose transcription level was significantly up-regulated in diseased fruit (Figure 5A). Since this effector was a small secreted protein, we named it CcSSP1. CcSSP1 homologs are ubiquitous in fungi and oomycetes and contain eight conserved cysteine residues (Supplementary Figure 4). In order to verify whether this effector could cause plant cell death, it was transiently expressed in the epidermis of *N. benthamiana* leaves by *Agrobacterium* infiltration. The results showed that CcSSP1 strongly caused cell death in *N. benthamiana* leaves (Figure 5B). Furthermore, recombinant CcSSP1 protein obtained from a prokaryotic expression system also induced cell death in *N. benthamiana* (Figures 5C,D). Additionally, we tested the transcript level of *NbPR1a* in *N. benthamiana* 2 days after transient expression of *CcSSP1*. Our results showed that CcSSP1 significantly activated the transcription of *NbPR1a* compared to a control GFP (Figures 5E,F). The transcription of *NbVSP2* was also significantly up-regulated, while the transcription of *NbPDF1.2* was suppressed (Figure 5G). This implied that *PDF1.2* and *VSP2* responded to JA signaling at different time levels. Correspondingly, the SA content of *N. benthamiana* also increased significantly after transient expression of *CcSSP1* (Figure 5H).

### CcSSP1 Interacts With the SA Signaling Marker MaPR1

**FIGURE 6:**
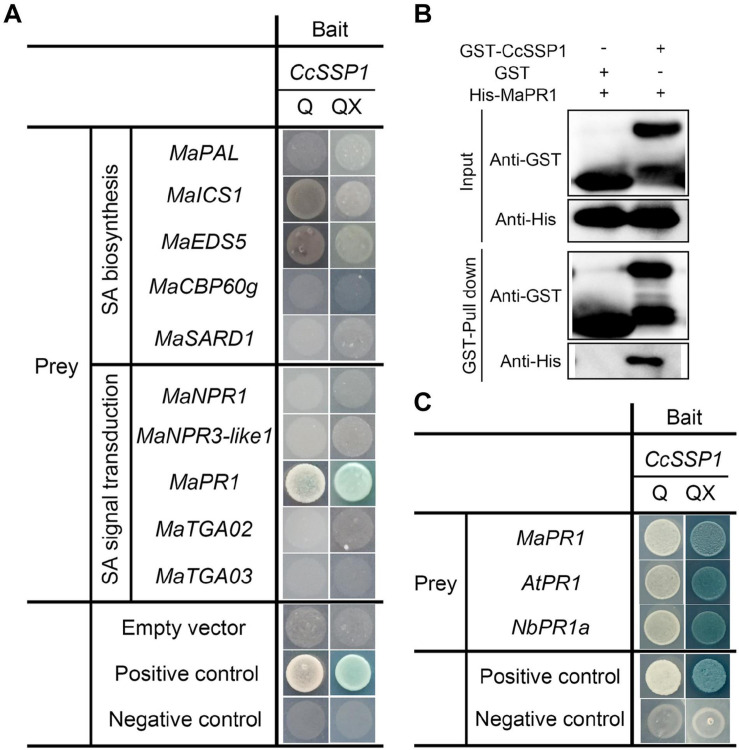
CcSSP1 interacts with MaPR1. **(A)** Y2H analysis of the interaction between CcSSP1 and key genes from the SA signaling pathway. pGBKT7-p53 was mated with pGADT7-T as a positive control, and pGBKT7-Lam was mated with pGADT7-T as a negative control. Q, SD/-His/-Leu/-Trp/-Ade (quadruple dropout medium); QX, SD/-His/-Leu/-Trp/-Ade + X-α-Gal + 200 ng/ml AbA + 10 mM 3-AT. AbA, Aureobasidin A; 3-AT, 3-amino-1,2,4-triazole. **(B)** *In vitro* pull-down assays of CcSSP1 with MaPR1. GST-tagged CcSSP1 and His-tagged MaPR1 were expressed in *Escherichia coli*. Coprecipitation of His-MaPR1 with GST-CcSSP1 was visualized by Western blot before (input) and after affinity purification (pulldown) using glutathione magbeads. **(C)** CcSSP1 and PR1 homologs interact in yeast. AtPR1, PR1 in *Arabidopsis thaliana*; NbPR1a, PR1 in *Nicotiana benthamiana*.

Since CcSSP1 activates SA signaling, we considered that CcSSP1 directly targets members of the SA signaling pathway. Some key genes for SA biosynthesis (*PAL*, *ICS1*, *EDS5*, *CPB60g*, and *SARD1*) and SA signal transduction (*NPR1*, *NPR3-like1*, *PR1*, *TGA2*, and *TGA3*) were used as candidates to determine whether they interacted with CcSSP1. Yeast two-hybrid (Y2H) analysis was performed to examine the interactions of CcSSP1 with candidate proteins. The results showed that CcSSP1 only interacted with MaPR1 (Figure 6A). To confirm a direct physical interaction between CcSSP1 and MaPR1 *in vitro*, we carried out pull-down assays using recombinant GST-tagged CcSSP1 and His-tagged MaPR1 from *E. coli*. Our results also indicated an interaction between CcSSP1 and MaPR1 (Figure 6B). In addition, Y2H analysis showed that CcSSP1 also interacts with MaPR1 homologs in *Arabidopsis thaliana* and *N. benthamiana* (Figure 6C).

## Discussion

### PAs Are Conserved Antimicrobial Substances in Plants

The biosynthesis of flavonoids and phenylpropanoids constitutes two vital metabolic pathways related to plant defense. The phenolic natural products produced by these pathways play a role in protecting against ultraviolet radiation, mechanical wounding, and insect infestation (Li et al., 1993; Osier and Lindroth, 2001; Mellway et al., 2009). Another widely valued function of phenolic natural products in plants is defense against pathogenic microbes (Dixon and Paiva, 1995; Hammond-Kosack and Jones, 1996; Dixon, 2001; Dixon et al., 2002). For example, PAs and dihydroquercetin are resistant to *Fusarium* species in barley (Skadhauge et al., 1997). Rice naringenin was shown to inhibit *Xanthomonas* strain growth and spore germination of *Pyricularia oryzae* (Padmavati et al., 1997). The mechanisms of flavonoid resistance to pathogens may act through the following modes: 1. By cross-linking microbial enzymes; 2. By inhibiting microbial cellulases, xylanases, and pectinases; 3. Through chelation of metals necessary for enzyme activity; and 4. via formation of a hard, almost crystalline structure as a physical barrier against pathogen attack (Treutter, 2005). Many phenolic natural products exhibit broad-spectrum antimicrobial activity. The transcriptome data from this study showed that in diseased mulberry, many positively regulated genes in the biosynthetic pathway of flavonoids and phenylpropanoid were significantly up-regulated.

Proanthocyanidins are derivatives of flavonoids and are abundant in mature mulberries (Li et al., 2020). We found that PA biosynthesis pathways were activated, especially in stage 2 diseased mulberry. This implied that PAs may be involved in resistance to the pathogens that cause mulberry sclerotial disease. ANR and LAR are essential for PA biosynthesis (Dixon and Sarnala, 2020). Overexpression of *ANR* and *LAR* from mulberry in tobacco increased the accumulation of PAs and the precursors catechin and epicatechin. Such transgenic plants have enhanced resistance to necrotrophic *B. cinerea* (Xin et al., 2020). *In vitro* experiments showed that PAs, rather than catechin and epicatechin, inhibited the vegetative growth of *S. shiraiana*, another causal agent of mulberry sclerotial disease. PAs are thus ubiquitous in plants and endow plants with basic resistance against potential pathogens.

### SA Signaling Is a Double-Edged Sword Against Necrotrophic Pathogens

Salicylic acid, JA, and ethylene are three classical plant defense hormones (Pieterse et al., 2012). There is a general rule that SA works against biotrophic pathogens, and JA and ethylene act against necrotrophic pathogens (Glazebrook, 2005; Bari and Jones, 2009). Additionally, auxin, abscisic acid (ABA), cytokinins (CKs), and brassinosteroids have emerged as cellular signaling molecules that function in molding plant-pathogen interactions (Robert-Seilaniantz et al., 2011). In this study, our data showed that the SA signaling pathway was activated, while the antagonistic JA signaling pathway was inhibited in infected mulberry fruits. *C. carunculoides* and the other two pathogens of mulberry sclerotial disease are necrotrophs, and the inhibition of JA signaling promoted this disease. Compared with healthy mulberry, the content of SA in infected mulberry was significantly increased. Mulberry is a kind of multiple fruit. Sclerotial disease causing pathogens spread from one drupelet to an adjacent drupelet until the whole fruit is covered. The SA content of the initial infected drupelet increased sharply, and the same was true in adjacent uninfected drupelets. However, the SA concentrations of drupelets in the middle stage of this disease were reduced to a level similar to that of healthy fruit. Regardless of whether considering the initial infected drupelet, the adjacent healthy drupelet or drupelets in the middle stage of the disease, the SA signaling pathway marker gene *MaPR1* was significantly up-regulated, while the JA signaling pathway marker gene *MaVSP2* was significantly down-regulated. *MaPDF1.2*, another marker gene of the JA signaling pathway, was only up-regulated in the initial infected drupelet. This indicated that JA signaling had a positive impact when the pathogen was recognized by mulberry, but it was subsequently suppressed. The inhibition of JA signaling may be related to the activation of the antagonistic SA signaling, and may also be related to other pathogenic factors or toxins of pathogen.

The SA immune pathway has pleotropic impacts on pathogens, especially for necrotrophic pathogens. SA is closely related to spontaneous cell death (Bruggeman et al., 2015). In this study, injection of 2 mM SA caused cell death in tobacco. However, SA pretreatment of *A. thaliana* prevented HR activation by *P. syringae* effector (Devadas and Raina, 2002). The results of this study showed that SA treatment slowed down the spread of pathogen in diseased fruits. In addition, low concentration SA pretreated tobacco displayed limit infection by *B. cinerea*. One reason may have been that low concentrations of SA and JA (10–100 μM) induced a transient synergistic enhancement of JA-SA defense signaling (Mur et al., 2006). However, the antagonism of SA-JA occurred with the prolongation of the treatment time or an increase in the hormone concentration (Mur et al., 2006). Another key reason may have involved the fact that SA induces SAR. NPR1 (non-expresser of PR genes 1) acts as a transcriptional coactivator and a master regulator of SAR that is indispensable for the expression of antimicrobial *PR* genes and for broad-spectrum resistance to disease (Durrant and Dong, 2004; Fu and Dong, 2013). Although SAR requires the co-regulation of SA and NPR1, and the expression of *NPR1* also requires SA induction, while NPR1 prevents the excess accumulation of SA in SAR activated regions by negatively regulating *ICS1* (Zhang et al., 2010). The establishment of SAR (through SA pretreatment or pathogen infection) enhan ced the resistance to pathogens of different lifestyles, including necrotrophs. This was because SAR-activated tissues increased their content of PR proteins, which have broad-spectrum antimicrobial activity (Loon et al., 2006; Zhu et al., 2012). Another key reason was that SA induced NPR1 to form a multi-component condensates in the cytoplasm, which inhibits programmed cell death and promotes cell survival (Zavaliev et al., 2020). Cell survival and maintaining immune activity are beneficial for limiting the expansion of pathogens, especially necrotrophic pathogens. However, it is precisely the cytoplasmic localization of NPR1 that inhibits JA-response genes (Spoel et al., 2003). It is not known whether the inhibition of JA signaling by NPR1 is related to the formation of SA-induced NPR1 condensates (SINCs). For host adaptive necrotrophs, this broad-spectrum defense is not robust enough to resist pathogen infection.

### The Effector CcSSP1 From *C. carunculoides* Activates SA Signaling by Targeting PR1

Phytohormones are small molecules that play a pivotal role in regulating plant growth, development, regeneration and defense. The crosstalk between SA and JA signaling helps plants to stimulate different defense pathways according to the type of attacker (Robert-Seilaniantz et al., 2011). Many attackers secrete small molecule effectors into plant cells in order to establish infection. The attackers target the mutually antagonistic SA and JA signaling pathways, thereby disrupting the SA-JA balance. Generally, SA signaling triggers resistance against biotrophic and hemibiotrophic pathogens as well as the establishment of SAR, whereas JA and ethylene signaling activates resistance against necrotrophic pathogens and pests (Glazebrook, 2005; Howe and Jander, 2008; Robert-Seilaniantz et al., 2011). For example, the effector Cmu1 secreted by the biotrophic fungus *Ustilago maydis* interferes with SA biosynthesis in maize and promotes smut (Djamei et al., 2011). The *P. syringae* type III effector AvrPtoB targets NPR1 and represses NPR1-dependent SA signaling, thereby subverting plant innate immunity (Chen et al., 2017). Similarly, RxLR48, an effector in the oomycete *Phytophthora capsici*, also targets NPR1 to inhibit SA signaling (Li et al., 2019). In order to suppress JA signaling, many herbivores and necrotrophic pathogens have evolved strategies to activate SA signaling. there is a salivary effector that promotes whitefly performance by eliciting SA signaling (Xu et al., 2019). The necrotrophic pathogen *B. cinerea* not only secretes exopolysaccharide to activate SA signaling, but also produces phytotoxin botrydial to induce the host’s HR-like cell death in an SA-dependent manner (El Oirdi et al., 2011; Rossi et al., 2011). The necrotrophic pathogenic fungus *Cochliobolus victoriae* secretes the mycotoxin effector victorin through interaction with TRX-h5 (Thioredoxin-h5), which may regulate NPR1 and activate the NB-LRR protein LOV1 to cause cell death (Sweat and Wolpert, 2007; Lorang et al., 2012). *S. sclerotiorum* SsCP1 is a rare example of a necrotrophic pathogenic effector regulating plant SA signaling. Moreover, SsCP1 is important for virulence in *S. sclerotiorum* and activates the SA signaling and directly interacts with PR1 (Yang et al., 2018).

In this study, we identified a small secreted protein CcSSP1 whose transcription level was significantly up-regulated during host infection with the obligate pathogen *C. carunculoides* that cannot be cultured *in vitro*. CcSSP1 strongly induced cell death in *N. benthamiana*. The transient expression of CcSSP1 in tobacco promoted an increase in SA content and significantly activated the expression of *NbPR1a*. The transcription of *PDF1.2*, a marker gene of the JA signaling pathway, was also inhibited. It is possible that the interaction of CcSSP1 and PR1 activated SA signaling through an unknown mechanism.

Based on our findings, we hypothesize that the interaction between CcSSP1 and PR1 may be widespread. The CcSSP1 homolog of *S. shiraiana* also interacted with the PR1 homolog of different plants (including MaPR1, AtPR1, and NbPR1a) in yeast (data not published). Recently, homologs of CcSSP1 have become objects of interest to other groups. VmE02, a CcSSP1 homolog from the apple Valsa canker pathogen *Valsa mali*, serves as a novel pathogen-associated molecular patterns (PAMP) (Nie et al., 2019). Nie et al. (2020) identified a receptor-like protein RE02 (also known as NbCSPR) that recognizes VmE02 in *N. benthamiana*. However, RE02 (NbCSPR) is a Solanaceae-specific receptor-like protein, and there is no obvious homolog in *A. thaliana*, *Solanum lycopersicum*, or *Malus domestica*. In this study, we identified a novel small secreted protein CcSSP1 from necrotrophic *C. carunculoides*, which activated host SA signaling and inhibited JA signaling to promote infection, possibly by targeting the conserved and critical protein PR1. Our results indicated that small secreted proteins play an important role in the pathogenicity of host-specific necrotrophs.

## Data Availability Statement

The datasets presented in this study can be found in online repositories. The names of the repository/repositories and accession number(s) can be found below: https://bigd.big.ac.cn/gsa, CRA003673.

## Author Contributions

NH and ZL contributed to the conception and design of the work. ZL, LH, ZH, YL, and YX performed the experiments analyzed data. BM, ZL, and YX analyzed transcriptome data. ZL, BM, and NH wrote the manuscript. All authors contributed to the article and approved the submitted version.

## Conflict of Interest

The authors declare that the research was conducted in the absence of any commercial or financial relationships that could be construed as a potential conflict of interest.

## Footnotes

1 http://www.ncbi.nlm.nih.gov/BLAST/

2 http://www.ncbi.nlm.nih.gov

3 http://www.expasy.ch/sprot

4 http://www.genome.jp/kegg

5 http://www.ncbi.nlm.nih.gov/COG

## Supplementary Material

Supplementary Figure 1 Mulberry fruits for RNA-seq uninfected and infected with *Ciboria carunculoides*.

Supplementary Figure 2 Statistics analysis of the number of genes obtained by RNA seq. **(A)** The numbers of genes belonging to plants and fungi were identified in all samples. **(B)** Differentially expressed genes (DEGs) in mulberries infected with *Ciboria carunculoides* at three different stages.

Supplementary Figure 3 Kyoto Encyclopedia of Genes and Genomes (KEGG) pathway enrichment analyses of DEGs in stage 2 and stage 3. **(A)** Top 20 pathways of KEGG enrichment in stage 2. **(B)** Top 20 pathways of KEGG enrichment in stage 3.

Supplementary Figure 4 Amino acid sequence alignment of CcSSP1 and its homologs in different fungi and oomycetes. Oomycetes are shaded on a gray background. Conserved amino acid residues are shaded on a black background. The eight conserved cysteine residues are individually annotated.

Supplementary Figure 5 Representative pictures of long-fruit mulberry with different disease levels.

Supplementary Table 1 Primers used in this study.
