# Supplementary figures and images for: Ciboria carunculoides Suppresses Mulberry Immune Responses Through Regulation of Salicylic Acid Signaling
Source: Front Plant Sci. 2021 Apr 6;12:658590. doi: 10.3389/fpls.2021.658590 (PMC8057602; doi:10.3389/fpls.2021.658590)

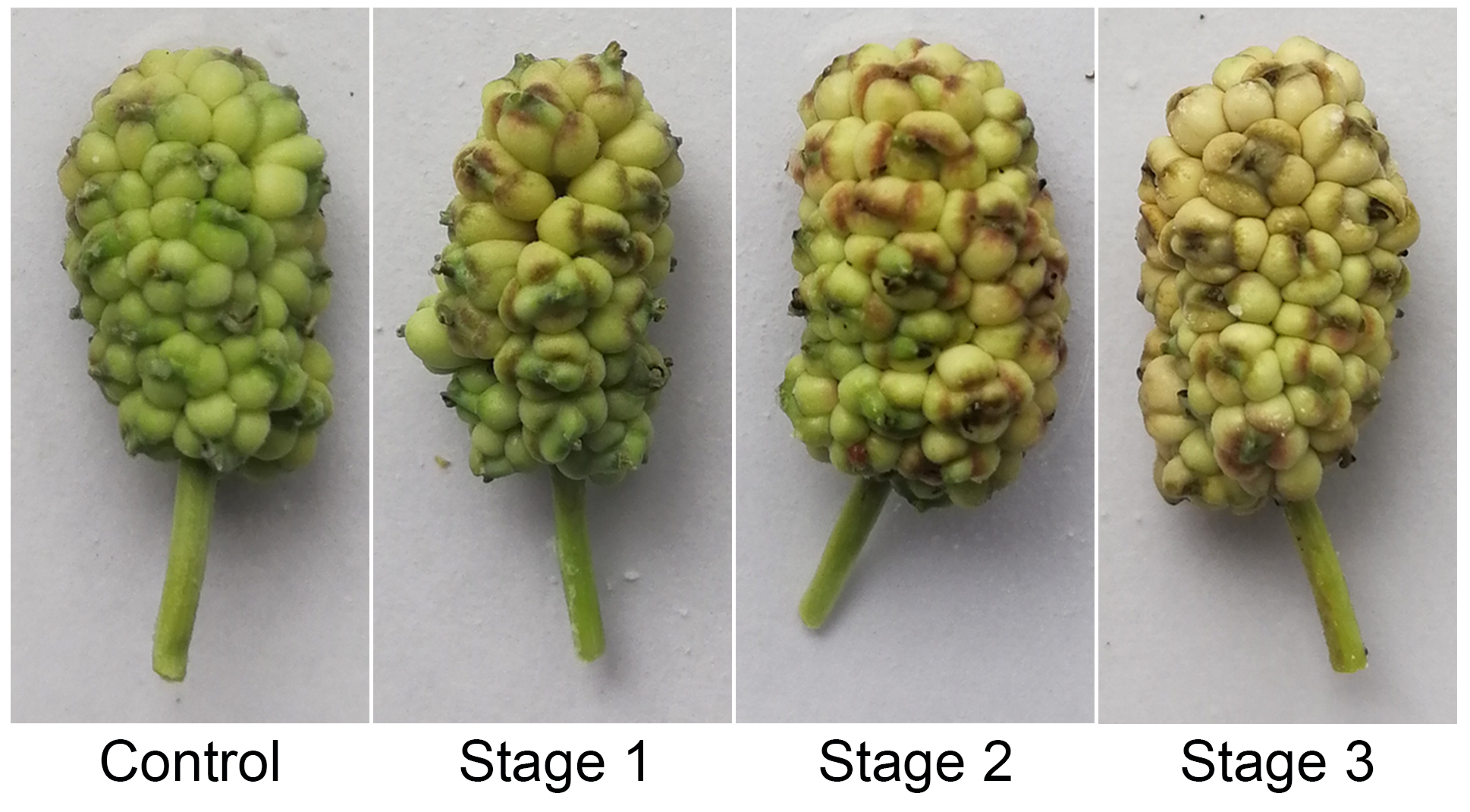

Supplement: Supplementary Figure 1 — Mulberry fruits for RNA-seq uninfected and infected with Ciboria carunculoides. [file Image_1.TIF]

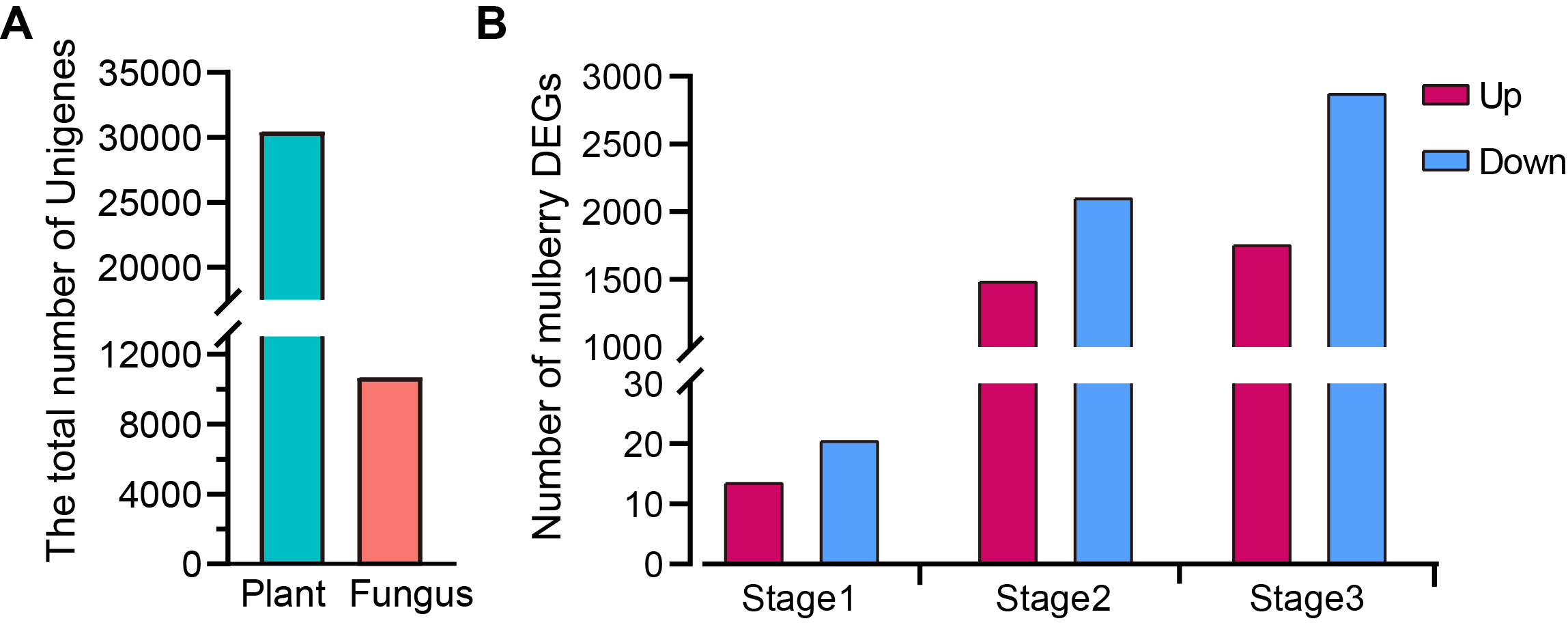

Supplement: Supplementary Figure 2 — Statistics analysis of the number of genes obtained by RNA seq. (A) The numbers of genes belonging to plants and fungi were identified in all samples. (B) Differentially expressed genes (DEGs) in mulberries infected with Ciboria carunculoides at three different stages. [file Image_2.TIF]

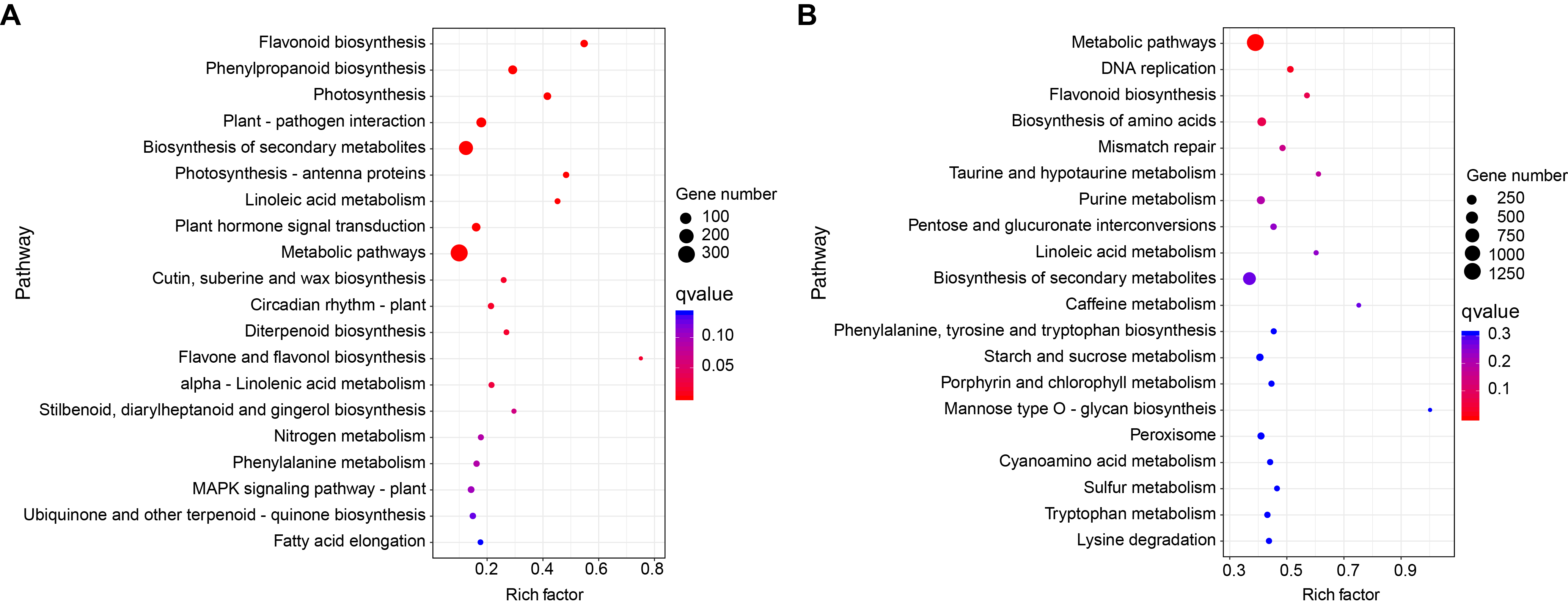

Supplement: Supplementary Figure 3 — Kyoto Encyclopedia of Genes and Genomes (KEGG) pathway enrichment analyses of DEGs in stage 2 and stage 3. (A) Top 20 pathways of KEGG enrichment in stage 2. (B) Top 20 pathways of KEGG enrichment in stage 3. [file Image_3.TIF]

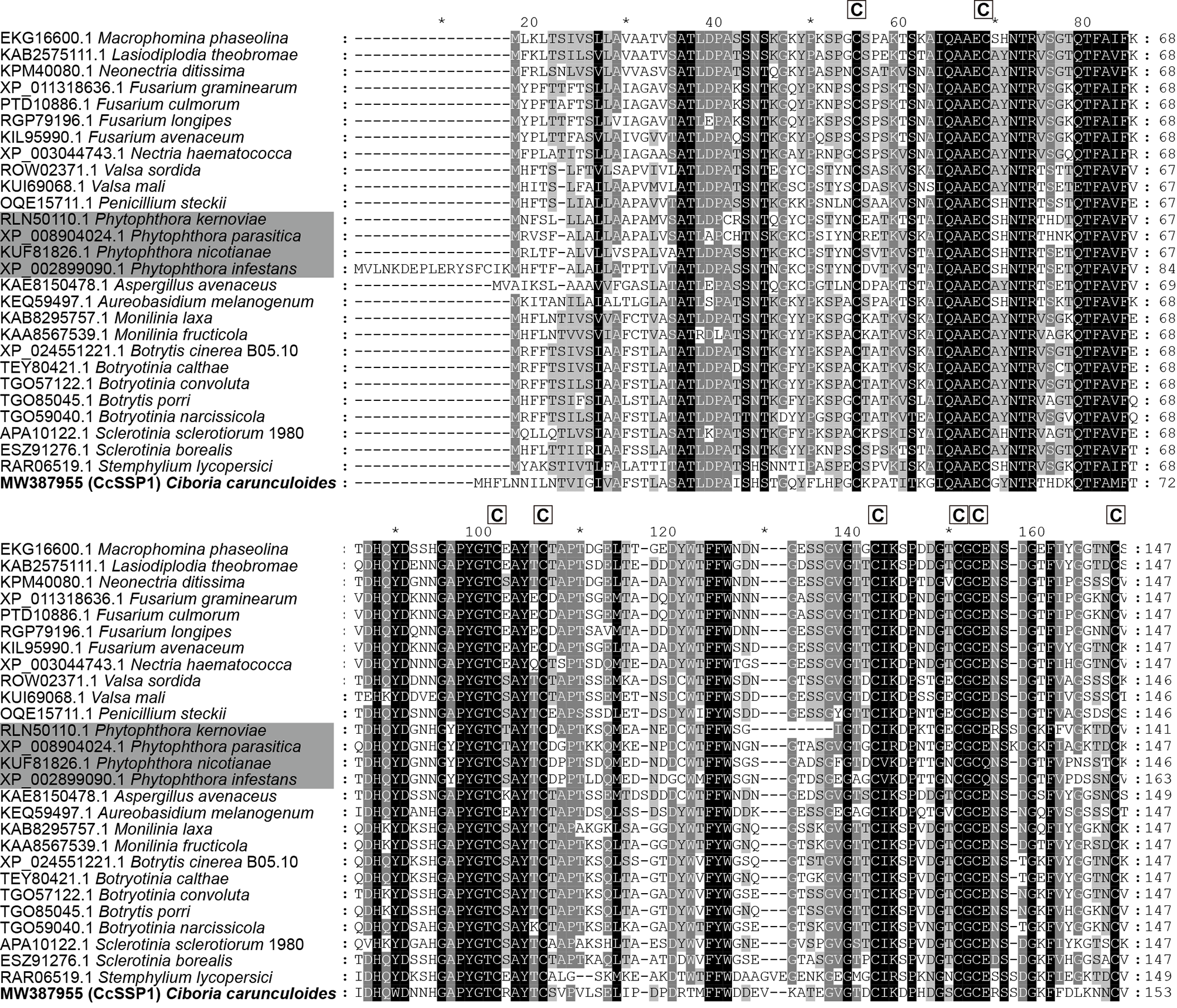

Supplement: Supplementary Figure 4 — Amino acid sequence alignment of CcSSP1 and its homologs in different fungi and oomycetes. Oomycetes are shaded on a gray background. Conserved amino acid residues are shaded on a black background. The eight conserved cysteine residues are individually annotated. [file Image_4.TIF]

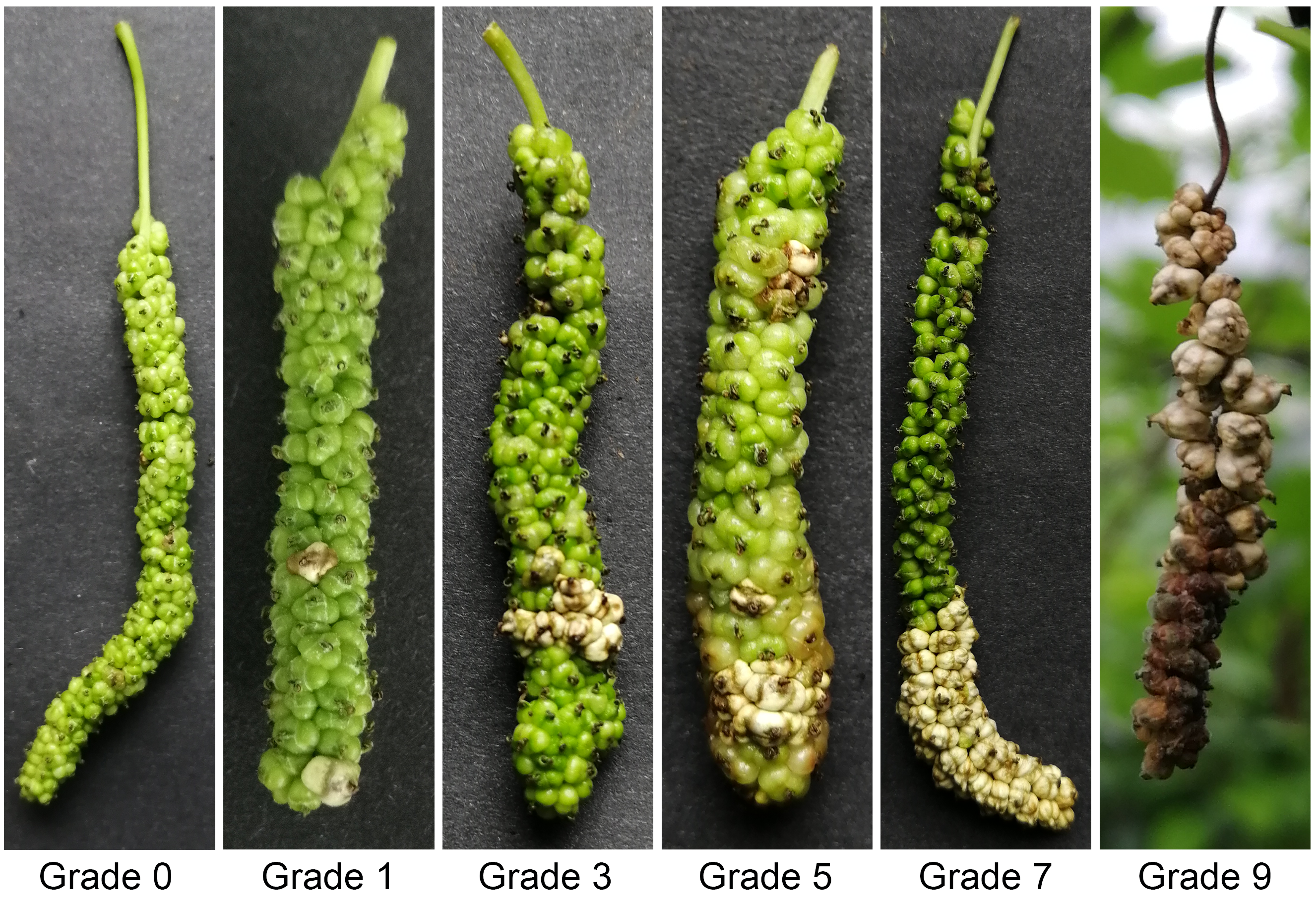

Supplement: Supplementary Figure 5 — Representative pictures of long-fruit mulberry with different disease levels. [file Image_5.TIF]
